# Brain age prediction using the graph neural network based on resting-state functional MRI in Alzheimer's disease

**DOI:** 10.3389/fnins.2023.1222751

**Published:** 2023-06-30

**Authors:** Jingjing Gao, Jiaxin Liu, Yuhang Xu, Dawei Peng, Zhengning Wang

**Affiliations:** School of Information and Communication Engineering, University of Electronic Science and Technology of China, Chengdu, China

**Keywords:** Alzheimer's disease, brain aging, resting-state functional magnetic resonance imaging, graph neural network, age prediction

## Abstract

**Introduction:**

Alzheimer's disease (AD) is a neurodegenerative disease that significantly impacts the quality of life of patients and their families. Neuroimaging-driven brain age prediction has been proposed as a potential biomarker to detect mental disorders, such as AD, aiding in studying its effects on functional brain networks. Previous studies have shown that individuals with AD display impaired resting-state functional connections. However, most studies on brain age prediction have used structural magnetic resonance imaging (MRI), with limited studies based on resting-state functional MRI (rs-fMRI).

**Methods:**

In this study, we applied a graph neural network (GNN) model on controls to predict brain ages using rs-fMRI in patients with AD. We compared the performance of the GNN model with traditional machine learning models. Finally, the *post hoc* model was also used to identify the critical brain regions in AD.

**Results:**

The experimental results demonstrate that our GNN model can predict brain ages of normal controls using rs-fMRI data from the ADNI database. Moreover the differences between brain ages and chronological ages were more significant in AD patients than in normal controls. Our results also suggest that AD is associated with accelerated brain aging and that the GNN model based on resting-state functional connectivity is an effective tool for predicting brain age.

**Discussion:**

Our study provides evidence that rs-fMRI is a promising modality for brain age prediction in AD research, and the GNN model proves to be effective in predicting brain age. Furthermore, the effects of the hippocampus, parahippocampal gyrus, and amygdala on brain age prediction are verified.

## 1. Introduction

Alzheimer's disease (AD) is a neurodegenerative disease commonly occurring in older adults (American Psychiatric Association and Association, 2013). People with AD usually first develop symptoms, such as mild memory degradation, followed by a continuous decline in cognitive function (Reitz et al., 2011), and are eventually diagnosed with AD after experiencing a brief phase of mild cognitive impairment (MCI). As the disease worsens, patients with reduced self-care ability become more dependent on family members for care, which seriously affects the patients' and their families quality of life. Therefore, the early diagnosis method for AD is of utmost importance. However, the existing diagnostic methods mainly rely on psychological tests and clinical observation for middle and advanced patients, lacking objectivity and effective early diagnosis methods. Therefore, it is essential to develop more accurate identification methods and find more objective biomarkers for early diagnosis, which will help patients receive treatment earlier and improve cure rates (Tahami Monfared et al., 2022; Warren and Moustafa, 2023). One such potential biomarker is brain age estimation, which could help detect mental disorders such as AD. Moreover, the neuroimaging-driven brain age prediction will contribute to studying the effect of AD on brain structures and functional networks. Meanwhile, recent studies have also highlighted that the significant delaying of the progression of MCI to AD will reduce both the prevalence and cost of AD (Anderson, 2019). To this end, it is crucial to identify individuals with accelerated brain aging by brain age prediction for precise identification and intervention of AD. Therefore, identifying those individuals at risk of developing AD early through accurate and reliable brain age prediction will significantly pave the way for developing effective preventive strategies (Villemagne et al., 2013).

With the development of artificial intelligence and medical imaging technology, studies focusing on brain age estimation have increased in recent years (Frizzell et al., 2022). Most of these studies have used structural magnetic resonance imaging (MRI) data (Gaser et al., 2013; Sajedi and Pardakhti, 2019; Bashyam et al., 2020; Levakov et al., 2020; Lee et al., 2022). For example, Bashyam et al. trained DeepBrainNet with two-dimensional images obtained by T1-weighted MRI with minimal preprocessing steps from 11,729 health control (HC) subjects. They achieved accurate age prediction and revealed that a moderately fitted model of brain aging was more suitable for distinguishing between AD and HC. This proposed method ensured the broad applicability of the model in clinical settings through straightforward preprocessing steps. However, a significant limitation is that it does not identify the anatomical regions affecting the model's performance. Lee et al. (2022) suggested that occlusion sensitivity analysis enhanced the interpretability of the model. They further discovered that the sulci and white matter were positively correlated with the brain age gap. In contrast, the gyri and periventricular regions were negatively correlated with the brain age gap. By conducting these analyses, the authors shed light on the effects of AD on brain structures. However, it is essential to note that structural changes in hippocampal atrophy occur many years after the accumulation of beta-amyloid (Aβ) pathology in the brain (McKhann et al., 2011; Villemagne et al., 2013), and impairment of functional connections in AD can be detected almost synchronously with Aβ and tau measured using positron emission tomography (PET). Therefore, detecting brain functional changes from resting-state functional MRI (rs-fMRI) could be a more sensitive and earlier method for individuals at risk of AD compared to the brain structural changes (Gonneaud et al., 2021). Previous studies have also demonstrated the utility of rs-fMRI data in predicting brain age and proposed that AD leads to accelerated aging. One such study adopted a Gaussian process regression (GPR) and obtained a mean absolute error (MAE) of 8.195 and a root mean square error (RMSE) of 10.31 in the test set (Millar et al., 2022). However, most existing studies using fMRI data for brain age prediction rely on traditional machine learning methods, and only a few studies apply deep learning networks. To the best of our knowledge, no study employs deep learning models for age prediction trained on graph data derived from functional connectivity (FC) and further applies those for diagnosing AD.

To summarize, while accelerating aging has been extensively investigated as the biomarker in the diagnosis of AD, many unknown aspects still exist for further exploration. Most studies have focused on structural MRI, which reveals structural changes in the brain. However, studies have shown that the fMRI change occurs earlier than structural changes, and resting state FC may be a more sensitive way to detect brain changes in preclinical AD patients (Gonneaud et al., 2021). Furthermore, while most studies using rs-fMRI have employed machine learning models, deep learning models may be more suitable for learning non-linear relationships in brain imaging data (Abrol et al., 2021). Meanwhile, the machine-learned feature may be more informative than those extracted by experience-based artificial methods.

To address these concerns, we have developed an attention-based graph neural networks (GNN) framework to detect accelerated brain aging in patients with AD. First, we extracted a Pearson correlation matrix from rs-fMRI and constructed graph data. Then we trained the GNN model on the graph data to predict brain ages in the HC group. Meanwhile, we applied the model to predict brain ages in patients with MCI and AD. Eventually, the results of our study were compared with those obtained from traditional machine learning models. In this study, we hope to make early diagnosis possible by identifying people who are likely to develop AD as early as possible.

## 2. Materials and methods

### 2.1. The dataset

The rs-fMRI data used in our study were obtained from the Alzheimer's Disease Neuroimaging Initiative (adni.loni.usc.edu) (ADNI) (Weiner et al., 2010). The ADNI is a multi-site longitudinal data repository, allowing researchers to access publicly available data upon request and approval, making a significant contribution to promoting the research related to AD. The repository contains a large amount of MRI, PET, and other medical image data. In addition, clinical, genomic, and biomarker data are also provided. Our study used the first available rs-fMRI images obtained during the medical follow-up period of 1,006 subjects from 48 sites, including 535 patients and 471 normal controls (ages 51–97 years). The specific demographic information is shown in Table 1.

**Table 1:** Demographic information of the subjects.

**Group**	**HC**	**SMC**	**EMCI**	**MCI**	**LMCI**	**AD**
Number	471	69	123	190	63	90
Age range	51–95	63–89	56–91	55–97	57–88	55–87
Mean age ± STD	72.13 ± 8.40	74.81 ± 5.77	73.50 ± 7.02	73.40 ± 8.34	73.37 ± 7.74	74.02 ± 7.55
Sex (M/F)	183/288	28/41	61/62	98/92	38/25	54/36

HC, healthy control; SMC, significant memory concern; EMCI, early mild cognitive impairment; MCI, mild cognitive impairment; LMCI, late mild cognitive impairment; AD, Alzheimer's disease; M, male; F, female; STD, standard deviation.

### 2.2. Data preprocessing

All the rs-fMRI images in the Digital Imaging and Communications in Medicine (DICOM) format were preprocessed using the Brainnetome fMRI Toolkit (Xu et al., 2018). The preprocessing steps include DICOM to Neuroimaging Informatics Technology Initiative (NIFTI) conversion, the deletion of the first 10 unstable time points, slice timing correction, registration, normalization to MNI standard space, and denoising. Then, based on the automatic anatomical labeling (AAL) atlas, which divides the human brain into 116 regions (Tzourio-Mazoyer et al., 2002), we extracted the time-series data from 116 brain regions and calculated Pearson correlation coefficients between two brain regions to obtain the Pearson correlation matrix. It is worth noting that the length of the time-series data was different because of different instruments of image acquisition. However, the calculation of the Pearson correlation matrix was not affected. Furthermore, we used Combat (Pomponio et al., 2020) on multi-site data to eliminate site differences. The ablation experiment was performed to verify the effect of Combat.

### 2.3. Graph data construction

In graph theory, a graph is generally denoted by a pair **G** = (**V**, **E**), where **V** refers to the vertex set and **E** represents the edge set. In this study, the FC matrix of each subject was constructed as graph-structured data. Specifically, 116 brain regions defined by the AAL atlas were assigned a corresponding vertex in the graph. The edges in the graph corresponded to the connections between brain regions. The FC values were treated as the features of vertices, while the FC values satisfying a threshold criterion were considered as the features of edges. A complete graph was obtained when the threshold was set to 0. Otherwise, edges that did not meet the threshold requirement were removed from the graph.

### 2.4. Brain prediction model based on the graph neural network

This study concerns the modeling of FC matrices as graph data, which characterizes the spatial correlations between diverse brain regions, as exemplified by Ktena et al. (2017). In this context, GNN is a prevailing method employed for feature learning of graph data by aggregating information from neighboring regions through convolution, thus displaying remarkable performance in graph representation learning (Zhang et al., 2019).

**Figure 1:**
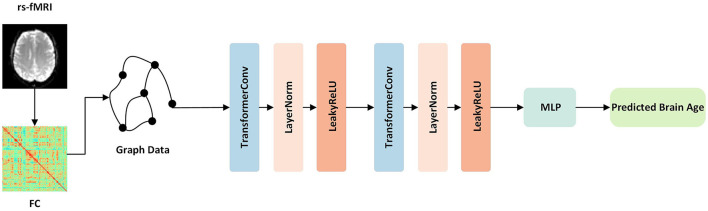
Overview of our proposed framework. First, the function connection matrices were extracted from the rs-fMRI data. Then, the graph data for each subject was constructed and fed into the network. The network contained TransformerConv layers, which updated the aggregated node features of the graph data. Finally, following one layer of MLP, the output was the predicted brain age.

This study introduces a novel approach for brain age prediction using a GNN model based on transformer convolution (TransformerConv) (Shi et al., 2020). The proposed framework was trained and evaluated on the ADNI dataset, and the graphical representation of the model is depicted in Figure 1. First, the TransformerConv layer was utilized to aggregate and update the node features of the graph data. To be more specific, the input vectors from the graph data were fed into the self-attention layer and calculated the query, key, and value for each region. Then, the self-attention coefficient was calculated, which represented the similarity between the query and the keyword. Finally, the self-attention coefficient was used as the weight, and the output vector of the layer was the weighted sum of the value. Then, the LayerNorm layer (Ba et al., 2016) was applied to normalize the neurons in the middle layer, ensuring distribution stability. The following LeakyReLU (Maas et al., 2013) activation layer was used to enhance the learning ability of the model via non-linear mapping. Finally, the outputs of the two layers mentioned above were used as inputs to a multilayer perceptron (MLP) consisting of a fully connected layer, ReLU layer (Glorot et al., 2011), BatchNorm (Ioffe and Szegedy, 2015), and another fully connected layer, with 32 hidden nodes and one output node, to predict the brain age.

We employed the deep learning approach for predicting age based on graph data from the HC group. Specifically, we utilized a batch size of 16, an initial learning rate of 0.001, and a weight attenuation of 0.001 with the Adam optimizer (Kingma and Ba, 2014) and the cosine annealing learning rate adjustment algorithm. Then, the model was trained based on the 10-fold cross-validation and the mean square error cost function as the loss function. The model's performance was evaluated by three criteria: MAE, RMSE, and the Pearson Correlation Coefficient (PCC) between predicted and chronological age. In addition, we compared our approach to the other six regression methods, namely, support vector regression (SVR) (Vapnik, 1998), GPR (Williams and Rasmussen, 1995), random forest regression (RFR) (Cutler et al., 2012), least absolute shrinkage and selection operator (LASSO) regression (LR) (Tibshirani, 1996), AlexNet (Krizhevsky et al., 2017), and autoencoder (AE) (Heinsfeld et al., 2018).

### 2.5. Estimated age difference in the patient group

We applied the model to analyze independent test samples of individuals categorized into distinct groups, including significant memory concern (SMC), early mild cognitive impairment (EMCI), MCI, late mild cognitive impairment (LMCI), and AD groups. Additionally, the brain age gap (BAG) as the difference between model-predicted and chronological age was calculated to investigate the potential impact of AD on the brain function.

### 2.6. *Post hoc* model based on perturbation

Deep learning is widely used in the diagnosis and treatment of AD. However, its lack of transparency hinders its clinical application as doctors need to understand the impact of changes in FC on patients with AD (Ahmedt-Aristizabal et al., 2021). GNN explainability methods have emerged as a promising solution to address this issue. In recent years, GNN explainability methods have developed rapidly. Existing methods can be divided into two categories: instance-level methods and model-level methods. The former includes the gradient/feature-based, perturbation-based, decomposition, and surrogate methods (Yuan et al., 2022), and the latter includes XGNN (Yuan et al., 2020). In this study, we used the *post hoc* model based on perturbation GNNExplainer (Ying et al., 2019) to explain networks and help doctors analyze pathology images related to AD. First, the features of each individual graph were perturbed, and the generated node feature mask was multiplied with the input graph element by element to obtain the masked graph. Then, the masked graph was used as the input of the trained model to evaluate and train the mask. The loss function was defined as the mean square error of predicted brain ages using the original graph and masked graph. When nodes with important information were retained, there was little change between the output before and after the perturbation, which would reveal the significant brain regions in the prediction task. Finally, we obtained the explainable weight matrix of each subject. The rows and columns represented the brain region corresponding to the node and the explainable weight of the brain region.

## 3. Results

### 3.1. Results of age prediction based on FC

This study evaluated a brain age prediction model based on GNN using a 10-fold cross-validation technique. A total of 471 data from the ADNI dataset were randomly divided into 10 groups for cross-validation. The test set comprised one group of data, while the remaining nine groups served as the training set, which was repeated 10 times. The performance of the model was assessed using the mean and standard deviation of the results obtained from 10-fold cross-validation. The results demonstrated that the model could effectively predict brain age, with an MAE of 5.92 ± 0.62, RMSE of 7.56 ± 0.78, and PCC of 0.44 ± 0.11.

Meanwhile, the results using the data before and after performing the Combat operation were compared. We also presented a comparative study of our GNN-based brain age prediction model against the other six methods (i.e., SVR, GPR, RFR, LR, Alexnet, and AE). It is important to note that traditional machine learning models utilize a one-dimensional vector as input. Thus, we flattened the upper triangular region of a symmetric 116^*^116 matrix by row, excluding the main diagonal. We then fed a one-dimensional vector with 6,670 features for each subject into the traditional models. Our results in Table 2 indicate that our GNN-based model outperformed other models in terms of MAE and RMSE, and the Combat operation enhanced the performance of the model. Furthermore, to ensure the consistency of different parameters, we also conducted experiments to investigate the influence of the number of heads on model performance. The results presented in Table 3 indicated that the model with a single head achieved the best performance. Additionally, we compared the performance of models with different numbers of self-attention layers, ranging from 1 to 4. The results, shown in Table 4, indicated that the model with two self-attention layers performed the best.

**Table 2:** Comparison with other methods.

**Method**	**MAE**	**RMSE**	**PCC**
SVR	6.38 ± 0.72	7.90 ± 0.70	0.39 ± 0.08
GPR	6.55 ± 0.72	8.24 ± 0.80	0.40 ± 0.11
RFR	6.14 ± 0.65	7.59 ± 0.59	0.43 ± 0.11
LR	6.03 ± 0.65	7.46 ± 0.64	**0.47** **±0.10**
Alexnet	5.98 ± 0.59	7.57 ± 0.72	0.42 ± 0.10
AE	7.65 ± 1.04	10.04 ± 1.07	0.25 ± 0.14
Our proposed model using data before the Combat	6.68 ± 0.52	8.48 ± 0.72	0.35 ± 0.13
Our proposed model using data after the Combat	**5.92** **±0.62**	**7.56** **±0.78**	0.44 ± 0.11

SVR, support vector regression; GPR, Gaussian process regression; RFR, random forest regression; LR, least absolute shrinkage and selection operator (LASSO) regression; AE, autoencoder; MAE, mean absolute error; RMSE, root mean square error; PCC, Pearson Correlation Coefficient.

**Table 3:** Comparison of the performances of models with different numbers of heads in the self-attention layer.

**Number of heads**	**MAE**	**RMSE**	**PCC**
1	**5.92** **±0.62**	**7.56** **±0.78**	**0.44** **±0.11**
2	6.89 ± 0.31	8.73 ± 0.51	0.31 ± 0.16
4	6.92 ± 0.48	8.71 ± 0.61	0.34 ± 0.14
8	7.09 ± 0.58	8.96 ± 0.54	0.33 ± 0.11

**Table 4:** Comparison of the performances of models with different numbers of self-attention layers.

**Number of self-attention layers**	**MAE**	**RMSE**	**PCC**
1	7.07 ± 0.61	9.49 ± 0.72	0.30 ± 0.14
2	**5.92** **±0.62**	**7.56** **±0.78**	**0.44** **±0.11**
3	6.70 ± 0.79	8.48 ± 0.93	0.34 ± 0.12
4	6.95 ± 0.52	8.97 ± 0.79	0.28 ± 0.14

### 3.2. Accelerated functional brain aging in patient groups

Our study aimed to investigate the relationship between BAG, defined as the difference between predicted and chronological age, and the diagnosis of AD using an independent test set comprising five groups of data, including SMC, EMCI, MCI, LMCI, and AD. We calculated criteria and BAG and analyzed the results using various statistical measures such as MAE, RMSE, and PCC. The findings of our study indicated a significant increase in the values of MAE, RMSE, and BAG in the AD group compared to the HC group, accompanied by a decrease in the PCC value, suggesting accelerated brain function aging in AD patients. Thus, BAG could serve as a valuable biomarker to evaluate the severity of AD and facilitate early diagnosis. The detailed results of each data group are summarized in Table 5.

**Table 5:** Results in HC, SMC, EMCI, MCI, LMCI, and AD groups.

**Group**	**MAE**	**RMSE**	**PCC**	**BAG**
HC	5.92 ± 0.62	7.56 ± 0.78	0.44 ± 0.11	−0.07 ± 1.25
SMC	5.96 ± 0.55	7.18 ± 0.58	0.14 ± 0.07	−1.39 ± 1.52
EMCI	5.99 ± 0.56	7.55 ± 0.68	0.29 ± 0.08	−0.14 ± 1.48
MCI	6.86 ± 0.48	8.77 ± 0.62	0.26 ± 0.06	−0.46 ± 1.26
LMCI	6.57 ± 0.47	8.35 ± 0.56	0.23 ± 0.09	−0.41 ± 1.44
AD	6.72 ± 0.53	8.14 ± 1.14	0.19 ± 0.08	0.06 ± 1.66

### 3.3. The explainability of our model

**Figure 2:**
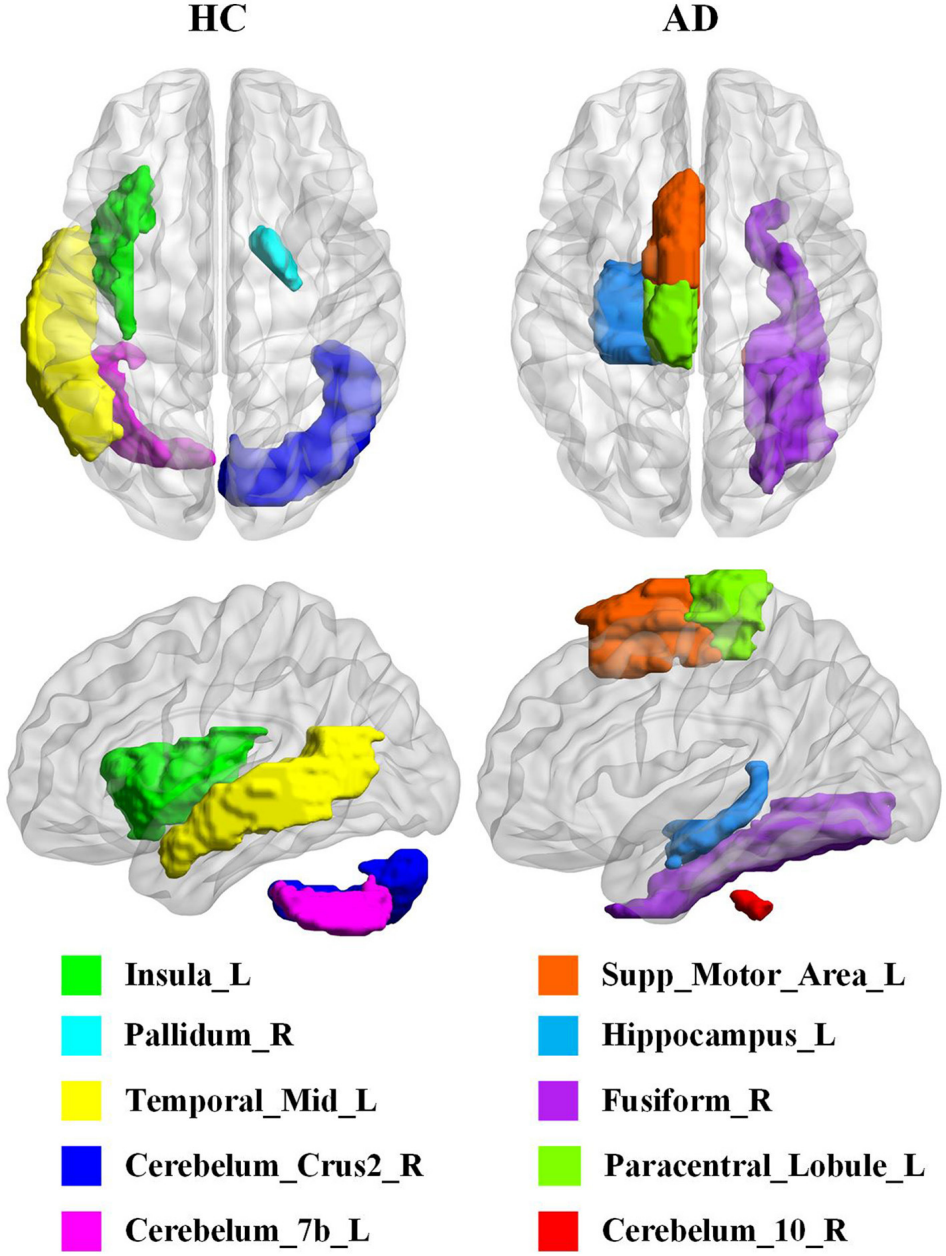
Visualization of the difference between HC and AD in the top 35 brain regions. Insula_L, Insula, left; Pallidum_R, Lenticular nucleus, pallidum, right; Temporal_Mid_L, Middle temporal gyrus, left; Cerebelum_Crus2_R, Cerebelum_Crus2, right; Cerebelum_7b_L, Cerebelum_7b, left; Supp_Motor_Area_L, Supplementary motor area, left; Hippocampus_L, Hippocampus, left; Fusiform_R, Fusiform gyrus, right; Paracentral_Lobule_L, Paracentral lobule, left; Cerebelum_10_R, Cerebelum_10, right.

To account for the neural substrates that impact the age prediction model and to elucidate functional changes that underlie deviations in brain age from typical aging trajectories in individuals with AD, we ranked the average explanatory weight coefficients in the HC and AD groups, respectively, and subsequently found the shared and distinct brain regions. We specifically focused on the top 30% of shared regions, which were primarily located in the medial and paracingulate gyrus, inferior and superior temporal gyrus, transverse temporal gyrus, anterior cingulate and paracingulate gyrus, anterior central gyrus, thalamus, and other brain regions, all of which made notable contributions to age prediction. Additionally, we observed that functional areas implicated in the diagnosis of AD, including the parahippocampal gyrus and amygdala, were also represented among these shared regions. These findings are reported in Table 6. Furthermore, our results revealed five distinct brain regions between the two groups shown in Figure 2 visualized using the BrainNet Viewer (Xia et al., 2013). Specifically, the HC group mainly focused on the inferior middle temporal gyrus, insula, globus pallidum, and two regions of the inferior cerebellum, which were not present in the AD group. In contrast, the AD group mainly focused on the inferior paracentral lobules, supplementary motor areas, fusiform gyrus, inferior cerebellum, and hippocampus, which were not observed in the HC group.

**Table 6:** Top 35 brain regions with their weights in HC and AD.

**Region name (HC)**	**Weight (HC)**	**Region name (AD)**	**Weight (AD)**
Median cingulate and paracingulate gyri, right	0.5232	Inferior temporal gyrus, left	0.5212
Inferior temporal gyrus, left	0.5220	Heschl gyrus, right	0.5200
Heschl gyrus, right	0.5208	Precentral gyrus, right	0.5192
Anterior cingulate and paracingulate gyri, left	0.5208	Median cingulate and paracingulate gyri, left	0.5159
Superior occipital gyrus, left	0.5202	Median cingulate and paracingulate gyri, right	0.5158
Angular gyrus, right	0.5198	Superior temporal gyrus, left	0.5154
Median cingulate and paracingulate gyri, left	0.5194	Thalamus, right	0.5145
Precentral gyrus, right	0.5191	Superior occipital gyrus, left	0.5137
Thalamus, right	0.5171	Superior occipital gyrus, right	0.5136
Cerebelum_9, right	0.5171	Inferior frontal gyrus, triangular part, right	0.5125
Superior temporal gyrus, left	0.5169	Cerebelum_Crus1, left	0.5123
Inferior frontal gyrus, triangular part, right	0.5168	Inferior frontal gyrus, opercular part, left	0.5121
Inferior occipital gyrus, right	0.5167	Anterior cingulate and paracingulate gyri, left	0.5121
Parahippocampal gyrus, left	0.5166	Angular gyrus, right	0.5120
Cerebelum_Crus1, left	0.5159	Parahippocampal gyrus, left	0.5118
Parahippocampal gyrus, right	0.5142	Amygdala, right	0.5113
Superior occipital gyrus, right	0.5135	Cerebelum_9, right	0.5111
Cerebelum_8, left	0.5133	Thalamus, left	0.5094
Amygdala, right	0.5121	Postcentral gyrus, right	0.5086
Thalamus, left	0.5121	Inferior occipital gyrus, right	0.5085
Inferior frontal gyrus, opercular part, left	0.5115	**Paracentral lobule, left**	0.5073
Lingual gyrus, right	0.5092	Cerebelum_4_5, left	0.5072
Vermis_6	0.5087	Vermis_6	0.5065
Caudate nucleus, left	0.5085	Parahippocampal gyrus, right	0.5063
Temporal pole: superior temporal gyrus, right	0.5083	Temporal pole: superior temporal gyrus, right	0.5058
Cerebelum_4_5, left	0.5082	Lingual gyrus, right	0.5053
**Middle temporal gyrus, left**	0.5078	**Supplementary motor area, left**	0.5047
**Insula, left**	0.5075	**Fusiform gyrus, right**	0.5047
Postcentral gyrus, left	0.5070	Superior frontal gyrus, dorsolateral, right	0.5044
Cuneus, left	0.5049	Cerebelum_8, left	0.5041
Superior frontal gyrus, dorsolateral, right	0.5049	Postcentral gyrus, left	0.5034
Postcentral gyrus, right	0.5043	**Cerebelum_10, right**	0.5032
**Lenticular nucleus, pallidum, right**	0.5039	Cuneus, left	0.5029
**Cerebelum_7b, left**	0.5039	**Hippocampus, left**	0.5025
**Cerebelum_Crus2, right**	0.5038	Caudate nucleus, left	0.5012

## 4. Discussion

In this study, we proposed a prognostication model that leverages GNN augmented with an attention mechanism that can adeptly anticipate brain age. Compared with machine learning methods that employ one-dimensional features, our proposed model retains the topological data of brain regions in the rs-fMRI data, thereby fortifying its FC representation. Furthermore, the attention network manifests superior capability in extracting global features.

Recent studies have identified the accumulation of Aβ plaques and tau protein chain accumulation as pathological markers of AD that are strongly associated with accelerated brain aging (Gonneaud et al., 2021; Mecca and van Dyck, 2021). Although structural MRI is commonly used to detect brain atrophy in AD patients, rs-fMRI is more sensitive to preclinical brain changes that occur earlier in the disease progression (Gonneaud et al., 2021). In this study, rs-fMRI data were used to develop a model based on graph neural networks to predict brain age in HC and different groups of patients. The resulting model achieved relatively accurate predictions, with an MAE of 5.92 as reported in Tables 2–4. However, our study found that the accuracy of brain age prediction based on rs-fMRI data was generally lower than that of studies using structural MRI data. This finding is consistent with previous research indicating that structural MRI is usually more accurate in predicting brain age (Liem et al., 2017; Bashyam et al., 2020; Cole, 2020; Levakov et al., 2020; Dunas et al., 2021; Hwang et al., 2022; Lee et al., 2022). The reason is due to the dynamic nature of rs-fMRI, which captures functional aging changes that may occur earlier than structural changes in the normal population, leading to our predicted older brain age in the subjects.

In this study, we aimed to identify the most critical brain regions for predictive tasks and AD diagnosis by explaining the model. The regions of considerable interest in neuroscience include the cingulate cortex, hippocampal structure, precuneus, inferior temporal gyrus, angular gyrus, and pivotal brain areas involved in default mode networks. Additionally, the supraoccipital gyrus within visual networks, the anterior and posterior central gyrus within sensorimotor networks, as well as the thalamus within the limbic system and other brain regions have garnered considerable attention within the field (Yao et al., 2010; Lei et al., 2020; Ma et al., 2022; Han et al., 2023; Zhou et al., 2023). These regions are crucial for understanding various neural processes and their interconnectivity. In addition, the medial temporal lobe is a vital region implicated in brain senescence and cognitive deterioration in individuals with AD. Our investigation also validated the considerable impact of specific brain regions, including the hippocampus, parahippocampal cortex, and amygdala, on the predictive capacity of our model, as previously demonstrated by Hrybouski et al. (2023). Consistent with an earlier study by Libby et al. (2012) and Liu et al. (2016), our results emphasize the critical role of parahippocampal gyrus connectivity in memory function and its association with AD severity. Furthermore, a positive correlation between cognitive decline and reduced FC between the amygdala and specific brain regions was observed in the studies by Yao et al. (2013) and Yao et al. (2014). Previous studies have consistently shown that hippocampal structure and function changes are closely linked to core memory deficits in individuals with AD (Khazaee et al., 2017; Ibrahim et al., 2021). The middle temporal gyrus, fusiform gyrus, paraventral lobule, cerebellum, and auxiliary motor area have been identified as regions displaying functional alterations that may be associated with accelerated brain aging in individuals with AD (Frisoni et al., 2009; Yao et al., 2010; Brier et al., 2012; Eavani et al., 2018; Hojjati et al., 2019; Ibrahim et al., 2021). These findings validate the utility of our interpretation prediction model as a valuable tool for investigating the advancement of AD.

## 5. Conclusion

In this study, we introduced a GNN-based prediction model that utilized rs-fMRI data for brain age estimation. Our comparative experiments revealed that our proposed approach outperformed other machine learning and deep learning prediction methods. Additionally, we confirmed the potential of the BAG as a diagnostic marker for AD. Moreover, our *post hoc* interpretation of the model identified crucial functional brain regions that contributed to age prediction and investigated possible reasons for accelerated brain aging. We anticipate that our research will facilitate the early detection of AD.

## Data availability statement

Publicly available datasets were analyzed in this study. This data can be found here: https://adni.loni.usc.edu.

## Ethics statement

The studies involving human participants were reviewed and approved by Institutional Review Board. The patients/participants provided their written informed consent to participate in this study. Written informed consent was obtained from the individual(s) for the publication of any potentially identifiable images or data included in this article.

## Author contributions

All authors listed have made a substantial, direct, and intellectual contribution to the work and approved it for publication.
